# Diploma Venders

**Published:** 1870-03

**Authors:** T. Williams


### DIPLOMA VENDERS.—REPLY OF DR. T. WILLIAMS.

EDITOR CHICAGO MEDICAL EXAMINER:

*Dear Sir:*—My attention having been called to your article headed "Diploma Venders," and which has been copied into other medical journals, and calculated to injure me by conveying a false impression, I request you, as an act of simple justice, to publish the following explanation:

I have no authority to *confer degrees;* my charter does not grant any special privileges (being simply an act of incorporation for business purposes, purely medical). I have never claimed or exercised such privilege. There is no "imposition" about the business — no swindle. I am not a "Diploma Vender," having never sold, conferred, or offered to sell or confer any degree in my life. I claim that I have not been guilty of anything, in this connection, which any respectable and regular medical man might not do.

So much for what I do not do. Now, allow me to explain what I do offer to do—premising the remark that my collegiate agency is aside and distinct from the medical and other business of the institute.

The principal object of this agency is, not to confer degrees upon improper or incompetent persons, but to "give such information as is necessary, before entering upon a course of studies," etc., with a view "to taking *any* of the learned degrees;" in other words, to *secure students* for the different law, medical, theological, and other colleges for which I may act as agent, by selling them *scholarships* in the same; *not diplomas.* These are all regular, good schools, and the requirements for graduation in them are the usual ones.

"Many States having passed laws prohibiting physicians who are not graduates from practising medicine," etc., the necessity arises for them to qualify themselves according to the requirements of the law; and here is a chance for them to do so, either by receiving an honorary degree (if entitled to such an honor), or by attending a final course of lectures and graduating. Any of the respectable medical colleges will confer degrees upon physicians who, although they may not have attended two full courses of lectures, have had sufficient experience in the study and practice of medicine to compensate for the deficiency. Five years reputable practice, or one course of lectures and three years' reputable practice, are usually considered as equivalent to the usual two didactic courses of lectures. Many of our very best physicians in the West belong to this class; many having been in active practice for 15 or 20 years without having ever graduated. No one can doubt but that such a physician, in reality, is better qualified to receive the degree of M.D. than three-fourths of the raw students, graduated at the end of their "second course."

On such cases, I simply act as the applicant's *attorney* before *any school* he may wish to graduate at. He furnishes me his credentials—say his tickets to first course, and certificates from other physicians as to his character, ability, and standing in the profession, length of time practising, etc. These, I simply lay before the faculty. If they think favorably of the application, they furnish him tickets for a course of lectures; he attends it or not as he pleases, so that he presents himself at commencement, passes examination, and receives his degree. There is nothing improper or irregular in all this, and no "imposition," because nobody is imposed upon or deceived.

In some instances where the case is clear that the physician is meritorious, an old and highly respected practitioner, for instance, an honorary degree may be conferred by the proper authorities, the examination being either merely *pro forma*, or dispensed with altogether.

Whether the colleges allowed me a commission for every scholarship I may sell for them, or whether I get a fee out of the applicant, for attending to the business, are mere private business matters which concern no one outside.

But as I have explained the nature of my agency, you will clearly perceive that your article does me an injustice which you are in honor bound to correct, as I have never, in any way, been a party to obtaining diplomas for any one not entitled to the same; and in the card you quote, I especially call your attention to the words "legitimately conferred." There is no skull-duggery about it, nor forgery of diplomas, nor abuse of the privilege of conferring them, nor anything of the kind—everything is fair, square, and legitimate. I should regret very much to have anything to do with anything that was not.

Yours truly, T. WILLIAMS, M.D.

